# Claudins in Retinal Disease: Beyond Barrier Integrity to Signaling and Therapy

**DOI:** 10.3390/cells15050417

**Published:** 2026-02-27

**Authors:** Mohamed S. Selim, S. Priya Narayanan, Payaningal R. Somanath

**Affiliations:** 1 Clinical and Experimental Therapeutics, College of Pharmacy, University of Georgia, Augusta, GA 30912, USA; 2 Department of Research, Veterans Affairs Health Care System, Augusta, GA 30912, USA; 3 Vascular Biology Center, Augusta University, Augusta, GA 30912, USA

**Keywords:** tight junctions, blood–retinal barrier, claudins, retinopathy, retinal pigment epithelium, endothelial permeability

## Abstract

**Highlights:**

**What are the main findings?**

- Claudin family members exhibit compartment-specific expression and regulation across the inner and outer blood–retinal barriers.
- Disruption of Claudin-mediated tight junctions is a recurring feature across developmental, metabolic, inflammatory, and degenerative retinal conditions.

**What are the implications of the main findings?**

- Claudins provide a unifying molecular framework linking retinal barrier dysfunction to disease progression.
- Understanding claudin-specific regulation may inform future strategies to monitor or stabilize blood–retinal barrier integrity in retinal diseases.

**Abstract:**

The blood–retinal barrier (BRB) maintains neurovascular homeostasis by regulating solute and ion exchange between the retina and circulation. This selectivity depends on tight junctions (TJs), with claudin (Cldn) proteins forming the core structure that defines paracellular permeability. Distinct Cldn isoforms show cell-specific expression, with Cldn-5 predominating in the endothelial cells of the inner BRB and Cldn-19 is the signature Cldn in the retinal pigment epithelium forming the outer BRB. Disruption of these isoforms contributes to vascular leakage, inflammation, and neuronal loss across various ocular diseases. Cldn function in vascular homeostasis is multifaceted; barrier dysfunction does not always result from Cldn loss, as excessive expression or mislocalization, particularly of Cldn-5, can also impair BRB integrity. Cldns act as dynamic signaling hubs that respond to metabolic, oxidative, and mechanical stress and are regulated through VEGF, Wnt/β-catenin, and RhoA/ROCK pathways. This review summarizes current understanding of Cldn biology in retinal vascular regulation and highlights emerging therapeutic strategies aimed at stabilizing Cldn expression and junctional localization. Small molecules and blocking antibodies that enhance localization or prevent degradation are redefining barrier repair. Key questions remain regarding isoform specificity, inter-barrier communication, and systemic safety. Integrative omics and spatial imaging may reveal disease-specific Cldn signatures and guide molecular restoration of BRB integrity.

## 1. Introduction

The retina is a highly laminated neural tissue with tightly organized neuronal and glial layers [1,2,3]. To sustain its intense metabolic demands, the retina harbors a unique vascular structure coupled with a well-defined blood-neural interface known as the blood–retinal barrier (BRB) [4]. Mirroring the blood–brain barrier (BBB), the BRB is a dual-barrier system consisting of two distinct but complementary components. The outer BRB (oBRB) is formed by the retinal pigment epithelium (RPE) and regulates molecular exchange between the retina and the choriocapillaris [5]. The inner BRB (iBRB) is primarily established by endothelial cells lining the retinal capillaries, forming a selective barrier that regulates solute and macromolecule exchange between the circulation and retinal tissue [5].

At the core of these barriers lie tight junctions (TJs), multiprotein complexes that seal the paracellular space between cells and define tissue-specific permeability [4,6]. Their disruption, even if subtle, underlies many ocular diseases [7]. As gatekeepers of barrier integrity, TJ proteins regulate “tightness” and ion selectivity of blood-tissue barriers (BTB) [4,6]. These protein complexes comprise members of the claudin (Cldn) family, occludin, and junctional adhesion molecules (JAMs), all anchored by cytoplasmic scaffold proteins such as zonula occludens (ZO-1 to ZO-3) [4,8,9].

Among TJ components, Cldns play a dominant structural and functional role, dictating tissue-specific barrier properties through isoform-dependent regulation of paracellular permeability [10]. Occludin associates with these strands to regulate TJ stability and signal transduction, while ZO-1 and ZO-2 anchor Cldns and occludin to the actin cytoskeleton and coordinate intracellular signaling [6,11]. More than 27 claudin isoforms have been identified in humans, each exhibiting distinct expression patterns that contribute to barrier heterogeneity across tissues. Cldn-3, -4, -5, -7, -18, and -19 have been indicated in epithelial-barrier regulation in various tissues [12,13,14]. Many studies have established Cldn5 in endothelial cells across various vascular beds [7,15,16,17,18,19], including retinal endothelial cells [20,21,22]. Loss of Cldn-5 has been shown to compromise endothelial integrity, leading to vascular leakage, neuroinflammation, neuronal degeneration, and intracerebral hemorrhage, directly contributing to disease progression [23,24,25]. The composition and dynamic regulation of Cldns ultimately dictate whether the retina maintains homeostasis or transitions toward barrier failure under stress [4,26].

In the retina, Cldns are differentially expressed across multiple cell types, owing to their fundamental role in barrier maintenance [4]. Despite mounting evidence highlighting the importance of these molecules in maintaining barrier function, the regulation of Cldn expression and their exact contributions to retinal physiology and pathology remain enigmatic. Therefore, this review aims to synthesize current evidence on the expression, regulation, and function of Cldns in the retina, emphasizing their roles in retinal disease and their potential as therapeutic targets for preserving or restoring visual function.

## 2. Cldns: Overview and Classification

Cldns are small (∼23 kDa) integral membrane proteins that constitute the structural backbone of TJ [10]. The term “claudin” is derived from the Latin word “*claudere*”, meaning “to seal.” Since their discovery in 1998, Cldns have been recognized as the essential determinants of paracellular barrier properties across epithelial and endothelial tissues [8,27]. The human genome encodes at least 27 distinct Cldn isoforms (Cldn-1 to Cldn-27), while mice express 24 closely related orthologs, underscoring the high degree of evolutionary conservation among species [28]. Each Cldn isoform exhibits a tissue- and cell-type-specific expression pattern, allowing for precise regulation of paracellular selectivity and barrier strength according to physiological demands [29]. This molecular diversity explains why endothelial barriers in the brain and retina exhibit exceptionally high tightness (high resistance and low ion permeability), while epithelial barriers in organs such as the kidney or intestine permit selective transport of ions and solutes to support their specialized functions.

### 2.1. Structural Features of Cldn Family of Proteins

Structurally, Cldns comprise a conserved tetraspan configuration of four transmembrane α-helices, two extracellular loops (ECL1 and ECL2), and short cytoplasmic N- and C-terminal tails [30]. The first extracellular loop (ECL1) is typically longer (~50 residues) and contains charged amino acids that determine ion selectivity, whereas ECL2 contributes to junctional strand formation and the cis/trans interactions between adjacent cells [31]. The second ECL (ECL2), however, appears to play a more essential role in transcellular binding of the paracellular space [32]. Additionally, the C-terminal tail of the Cldns usually ends with a PDZ-binding motif that allows binding to cytoplasmic scaffold proteins such as ZO-1, ZO-2, and ZO-3, which connects Cldns to the actin cytoskeleton and coordinates their spatial organization within the junctional complex [33]. Mutational or post-translational changes in this PDZ-binding region can radically alter TJ stability and downstream signaling, emphasizing the dynamic role of Cldns in regulating cell polarity and communication [9,32,34,35].

### 2.2. Functional Classification of Cldns

Functionally, Cldns are categorized as barrier-forming or pore-forming proteins depending on their permeability status. Barrier-forming Cldns, also referred to as “sealing” or “tightening” Cldns, include Cldn-1, -3-9, -11-14, -16, -18, and -19. These Cldns effectively reinforce the junctional strands and are fundamental to the sealing function required by highly impermeable barriers, such as the BBB and the BRB. Conversely, pore-forming Cldns are considered “leaky” to cations and anions. The ‘cation pore’ forming Cldns include Cldn-2, -10b, and -15, and the ‘anion pore’ forming Cldns include Cldn-10a and -17 complexes [10,26]. These Cldns essentially function as ion-selective channels that allow controlled passage of small cations or anions, such as chloride and sodium [36,37]. These opposing roles are largely controlled by both the amino acid charge and distribution in extracellular loops; for example, Cldn-2 and Cldn-15 isoforms constitute cation-selective permeation, whereas Cldn-10b favors anion flux. Interestingly, multiple Cldns can co-express in the same tissue, mediating similar or varying functions. For instance, Cldn-16 and Cldn-19 form heteromeric complexes in the kidney to regulate Mg^2+^ and Ca^2+^ reabsorption [38]. Dynamic regulatory mechanisms, including phosphorylation, palmitoylation, and interactions with signaling pathways such as Wnt/β-catenin and TGF-β, can further modulate Cldns function and alter their localization or channel behavior [39,40,41].

### 2.3. Tissue-Specific Distribution of Cldn Isoforms

The tissue-specific distribution of Cldns dictates their specialized physiological roles. In epithelia, Cldn-1 and Cldn-4 protect the skin barrier against water loss [42], while Cldn-7 and Cldn-18 regulate ion fluxes in the lung and stomach [43,44]. Cldn-19 is particularly exclusive in the RPE, as mutations in this isoform derive familial hypomagnesemia with hypercalciuria and nephrocalcinosis (FHHNC), often presenting with optic atrophy and macular coloboma, highlighting its role in retinal homeostasis [45]. Several Cldns are expressed in the kidney in a segment-specific manner. For instance, Cldn-2 is abundant in the proximal tubules, where it facilitates cation and water permeability [46], while Cldn-10 is localized in the thick ascending limb of Henle’s loop and contributes to sodium transport [47]. In the cochlea, Cldn-14 plays a vital role in preserving ionic gradients within the endolymph [48]. Evidence from in vitro models, in vivo studies, and human genetic analyses further demonstrates that mutations in Cldn-14 are associated with hereditary deafness [49,50,51]. In endothelial cells, Cldn5 dominates, forming the structural base of both the BBB and BRB, warranting highly contained paracellular permeability [4,52]. Endothelial reduction in Cldn-25 and the ensuing hyperpermeability have been reported in cerebellar hemorrhage [53]. Oligodendrocytes also express Cldn-11, which maintains myelin sheaths and contributes to rapid axonal conduction [54]. The anion pore-forming Cldn-17 plays a key physiological role in maintaining electrolyte balance, reactive oxygen species homeostasis, and vascular integrity across the BTB [55]. Its deficiency, however, leads to electrolyte imbalance, oxidative stress, kidney injury, and increased vascular permeability with associated inflammation and lung injury, highlighting its importance in barrier function and immune regulation under both physiological and pathological conditions [56].

### 2.4. Physiological and Pathophysiological Implications of Cldns

Collectively, the Cldn family exemplifies how minor molecular variations can result in a vast range of physiological and pathological outcomes across tissues. Their regulatory plasticity further makes them indispensable for preserving compartmental homeostasis in various organ systems.

## 3. Retinal Expression of Cldns

**Figure 1:**
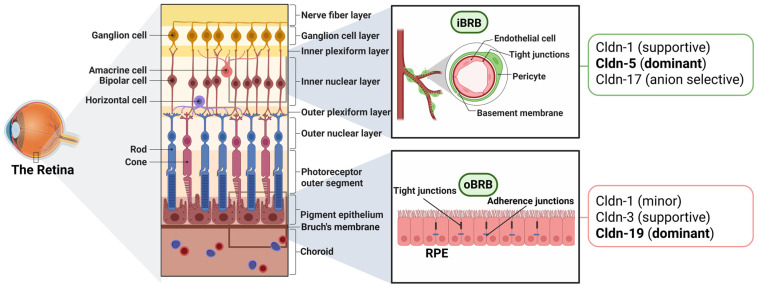
Structural organization of the retina and Cldn composition of the inner and outer blood–retinal barriers. Created in BioRender. Salah, M. (2026) https://BioRender.com/7mbgopz (accessed on 24 February 2026).

Owing to its cellular heterogeneity, the retina exhibits a vastly compartmentalized distribution of Cldns (Figure 1). Distinct Cldn isoforms localize to the retinal vascular endothelium, RPE, and, to a lesser extent, glial cells, ensuring selective permeability of both the oBRB and iBRB [1,57]. A range of methods, including immunohistochemistry, in situ hybridization, qPCR, RNA sequencing, and proteomic analysis of isolated endothelial or RPE cells, has been used to define Cldn expression patterns in retinal tissue [58,59].

### 3.1. Cldns in the Retinal Pigment Epithelium

The RPE is a distinct epithelium whose apical surface directly faces the outer retina, while its basal surface interacts with the underlying choriocapillaris to form the oBRB [60]. The oBRB is disrupted in retinal diseases such as AMD, in which choroidal capillaries breach the RPE monolayer and separate it from the photoreceptors. The leakiness of the choriocapillaris, due to junctional disruption, allows serum components to infiltrate the apical surface of the RPE, thereby perturbing retinal homeostasis [61].

In the human RPE, Cldn-19 is essential for normal development and retinal structure, and patients with nonfunctional Cldn-19 exhibit severe visual impairment due to developmental anomalies such as macular colobomata and significant myopia [45]. Beyond serving as a physical barrier, the RPE is also known for its critical phagocytic capacity, daily engulfing and degrading shed photoreceptor outer segments (POSs) through a tightly regulated process of ingestion, phagosome transport, maturation, and degradation [62]. Thus, impaired phagocytosis in the RPE is a central pathological feature of many macular degenerative diseases [63]. Cldn-19 appears integral to this phagocytic function, as its knockdown has been reported to hamper POS clearance and induce autophagic stress by upregulating SQSTM1 (p62), a marker of autophagy and cellular stress. This highlights the importance of Cldn-19 in maintaining RPE homeostasis and phagocytic activity [64] (Table 1).

Cldn-3 is also uniformly expressed in the RPE, though less abundantly than Cldn-19. Both Cldns have been shown to regulate RPE gene and protein expression independently of their TJ localization, suggesting additional roles in epithelial differentiation and homeostasis beyond barrier formation [65]. Cldn-1 and Cldn-10 are likewise expressed in the RPE, albeit at relatively low levels compared to Cldn-19, indicating that they may play supportive or region-specific roles rather than be a sealing component of the RPE barrier [66]. Altogether, Cldn-19 remains the principal structural and functional Cldn maintaining RPE barrier integrity.

### 3.2. Cldns in the Endothelial Cells of the iBRB

The iBRB is established by non-fenestrated endothelial cells that line the blood vessel lumen, supported by pericytes and astrocytic endfeet, and is particularly enriched in Cldn-5, which is critical for maintaining the selective permeability of retinal microvessels, making it a molecular gatekeeper of the iBRB [70,71]. In the retina, Cldn-5 co-localizes with Cldn-1, as studies using immortalized bovine retinal endothelial cells (iBRECs) demonstrated that Cldn-1 is an essential component of functional TJs in retinal endothelial cells. Further, prolonged exposure to vascular endothelial growth factor (VEGF) (over three days) markedly depleted Cldn-1 from the plasma membrane in iBRECs within 24 h, which strongly correlated with increased permeability. Cldn-5, however, exhibited only subtle alterations under the same conditions [69]. Supporting this regulatory role, overexpression of Cldn-5 in cultured human retinal endothelial and pigment epithelial cells reinforced barrier integrity by upregulating Cldn-1 and downregulating Cldn-2, shifting the balance toward a tighter, less permeable junction profile. These findings highlight the central role of Cldn-5 in regulating the interaction dynamics of other Cldns and coordinating retinal TJ assembly and vascular stability [75].

### 3.3. Cldns in Müller Cells and Other Retinal Cell Types

Müller glia, the most abundant glial cell type in the retina, constitute a crucial part of the neurovascular unit (NVU) [76]. Unlike endothelial or epithelial cells, Müller cells and astrocytes do not typically express canonical TJ proteins. Instead, they provide a pivotal structural and regulatory role in maintaining BRB integrity [73]. However, in the retina of teleost fish, Müller cell endfeet were reported to form TJs within the nerve fiber layer, expressing Cldn-1, -3, and -19, suggesting a specialized, species-dependent barrier role. In contrast, such Cldn expression is absent in mammalian Müller cells at the inner limiting membrane (ILM) [74]. Müller glia rather contributes to the formation of the outer limiting membrane (OLM), which contains TJ-associated proteins like occludin and ZO-1. Therefore, the OLM is regarded as a diffusion-limiting barrier rather than a classic TJ barrier [77].

Astrocytes are primarily located near the ILM and along the retinal vasculature. Together with pericytes, they ensheathe the retinal capillaries and serve as key regulators of the iBRB endothelial cells [78]. Due to the close interaction between astrocytic endfeet processes and endothelial cells, signaling factors released by the astrocytes can influence the regulation of Cldn expression in the endothelial cells [79]. In total, Müller cells and astrocytes generally do not express Cldns to form their own primary restrictive TJs in the mammalian retina.

### 3.4. Developmental Regulation of Cldns in the Retina

The development of a fully functional retina necessitates the precise coordination of numerous genes across defined spatial domains and developmental stages [80]. Barrier formation during this process can be traced through high-throughput expression profiling. For example, isolating RNAs from developing retinas of animal models like mice or zebrafish can reveal dynamic transcriptional changes over time [81]. Cldn expression is tightly regulated in a stage-dependent manner, making these developmental transitions highly sensitive to both genetic and environmental perturbations [79].

#### 3.4.1. Postnatal Cldn Expression Dynamics

During mouse retinal development, several Cldns, including Cldn-1, -2, -3, -4, -5, -12, -22, and -23, are developmentally regulated from postnatal day (P)8 to P21. Nevertheless, mRNA expression does not always translate into proper protein localization in the functional TJs. When the retinal vasculature was examined directly, only Cldn-1, -2, and -5 were actually incorporated into the TJs of endothelial cells, modulating the structure and integrity of the iBRB [57].

#### 3.4.2. Cldns in Neurovascular Signaling and Barrier Formation

The formation of retinal barriers is a dynamic process, not merely an intrinsic characteristic of the vasculature; it is influenced by inductive signals from the surrounding neural tissue [78]. For instance, Wnt signaling is recognized as a crucial requirement in orchestrating the formation of both the BBB and the BRB. Canonical Wnt ligands, particularly Wnt7a and Wnt7b, are secreted by the neuroepithelium and bind to Frizzled (FZD) receptors on endothelial cells, promoting physiological angiogenesis and mediating barrier growth via the Wnt/β-catenin signaling pathway [82]. The abundant expression of Cldn-5 in the retinal endothelium depends partly on this signaling cascade through a highly specific Norrin-Frizzled-4 (Fz4) signaling axis. Norrin is a high-affinity ligand that binds to the Fz4 receptor on endothelial cells. Loss or genetic disruption of any component of this pathway results in reduced Cldn-5 levels and increased retinal vascular permeability [82]. This specific interaction subsequently activates Wnt signaling, which is fundamental for both initiating and sustaining high levels of Cldn-5 within the retinal vasculature during the early postnatal retinal maturation stage in mice. Similarly, in humans, loss-of-function mutations in either FZD*4* or the Norrin gene (NDP) cause severe retinal hypovascularization syndromes, ultimately leading to blindness [83,84]. Although Cldn-5 levels have not been examined in human tissues, NDP-KO mice showed a significant reduction in Cldn-5 accompanied by increased expression of plasmalemma vesicle-associated protein (PLVAP), a marker of immature or fenestrated endothelium [85]. Such findings suggest the possibility that similar TJ deficits may occur in human FZD4/NDP-associated disease, although this remains to be experimentally investigated. Moreover, the integrity of the iBRB also exhibits circadian modulation, as Cldn-5 expression is regulated in a diurnal manner. In mice, retinal microvascular Cldn-5 levels are significantly lower in the evening compared to the morning [86]. In contrast, the development of the oBRB involves a distinct compositional shift in Cldn expression. Early in development, the RPE transiently expresses Cldn-5, which is then progressively replaced by dominant Cldn-19 as maturation proceeds, resulting in the establishment of a highly selective and restrictive epithelial barrier [68].

#### 3.4.3. Cldns in Stress Responses and Compensatory Regulation

Differential responses to cellular stress pathways also highlight the diverse regulation of the iBRB versus the oBRB. For example, in mouse retinal endothelial cells, endoplasmic reticulum (ER) stress downregulated Cldn-5 expression and contributed to vascular impairment [87]. In contrast, in RPE cell lines, ER stress promoted the upregulation of both Cldn-1 and occludin genes and protein expression, enhancing the trans-epithelial/endothelial barrier resistance (TEER) of the RPE cells [88]. This pattern suggests that the oBRB may attempt a compensatory tightening response under stress when the inner BRB fails early. This difference helps explain why vascular leakage is often the first detectable event in many systemic vascular disorders. Overall, retinal barrier integrity develops through coordinated neurovascular signaling, transcriptional control, and time-dependent gene expression. Defining these pathways improves our understanding of normal retinal development and mechanisms that drive barrier breakdown in ocular diseases.

## 4. Cldns in Retinal Diseases

### 4.1. Diabetic Retinopathy

Diabetic retinopathy (DR) is a major microvascular complication of diabetes mellitus (DM) that is characterized by chronic hyperglycemia-induced damage to the retinal capillary endothelium and supporting cells [89]. Globally, DR has emerged as one of the leading causes of vision loss among working-age adults. According to the International Diabetes Federation, around 536.6 million individuals were living with DM in 2021, a figure projected to rise to 783.2 million by 2045 [90]. Meta-analytic data indicates that 22.3% of the pooled global patients with diabetes exhibit clinical signs of DR [91].

DR is clinically categorized into two major stages: non-proliferative (NPDR) and proliferative (PDR). While NPDR represents the early stage, typically characterized by increased retinal vascular permeability and the appearance of microaneurysms and intraretinal hemorrhages, PDR is defined by severe pathological angiogenesis, with neovascularization and associated sequelae of vitreous hemorrhage or tractional retinal detachment (TRD) [92].

#### 4.1.1. Early BRB Dysfunction and Cldn Mislocalization

Disruption of TJ complexes in the iBRB, particularly through altered Cldn expression, is a defining feature of ischemic retinopathies (IRs), particularly in DR and retinopathy of prematurity (ROP), and similarly contributes to barrier instability in non-ischemic conditions such as age-related macular degeneration (AMD) [93,94,95]. Current therapeutic strategies, such as laser photocoagulation, anti-VEGF therapy, intravitreal corticosteroid injections, and vitreoretinal surgeries, can slow disease progression and, in some cases, partially improve vision. However, they do not uniformly restore barrier integrity and do not directly address the underlying molecular defects within TJ complexes [96,97,98,99]. Consequently, there is growing interest in Cldn-targeted interventions, including small molecules, RNA silencing, and pharmacological stabilizers to restore barrier integrity and prevent vision loss [22,100,101,102,103].

#### 4.1.2. Hyperglycemia, AGEs, and Post-Translational Regulation of Cldn-5

From a pathophysiological standpoint, persistent hyperglycemia triggers a plethora of endothelial-disrupting biochemical pathways, including oxidative stress, activation of the polyol and protein kinase C pathways, accumulation of advanced glycation end-products (AGEs), and dysregulation of growth factors such as VEGF [104]. Collectively, these mechanisms drive endothelial and pericyte dysfunction, basement membrane thickening, capillary occlusion, and eventual breakdown of the BRB [105]. Given this, the role of endothelial TJ proteins becomes central, and since the Cldn family constitutes the backbone of TJ strands, it is pivotal in maintaining paracellular permeability and barrier integrity [26]. In experimental preclinical DR, BRB disruption typically manifests as a diffuse mild leakage without significant edema formation, in contrast to the localized, profuse leakage that is observed during the advanced clinical stage of NPDR [106].

**Figure 2:**
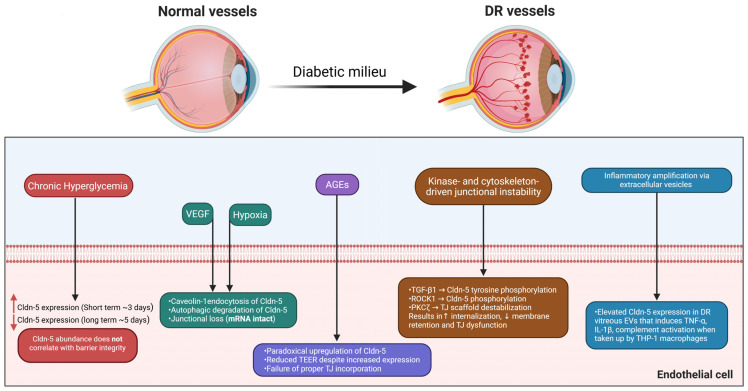
Mechanisms of Cldn-5 dysregulation in diabetic retinopathy. Created in BioRender. Salah, M. (2026) https://BioRender.com/wdo8i9t (accessed on 19 February 2026).

Among the endothelial TJ proteins, Cldn-5 is one of the most extensively studied Cldns in DR, and its altered expression, including loss, mislocalization, or upregulation, has repeatedly been shown to induce a complex and sometimes paradoxical impact on retinal vascular health (Figure 2). For instance, exposure of human retinal endothelial cells (HRECs) to hyperglycemic conditions (HG) for three days downregulated Cldn-5 and subsequently resulted in increased paracellular permeability [107]. In contrast, extended HG exposure for five days in the same cell line has been reported to upregulate Cldn-5 expression, suggesting a time-dependent adaptive or compensatory response [21]. Consistently, in the streptozotocin (STZ)-induced diabetic rat model, Cldn-5 expression was temporally reduced (at 6 weeks of diabetes), contributing to increased capillary leakage. This was further corroborated in VEGF-treated bovine retinal endothelial cells (BRECs) within 48 h, reinforcing its sensitivity to pro-permeability stimuli [108]. This discrepancy implies that the underlying defect is not just a lack of protein synthesis but rather a failure of post-translational control, stability, and junctional anchoring, which impedes proper Cldn-5 incorporation into the TJ strand, leading to barrier leakage despite elevated protein levels. Of relevance, certain endogenous factors appear to exert protective effects on endothelial junction integrity [109]. C1q/tumor necrosis factor-related protein-3 (CTRP3) has been identified as a regulator of BRB integrity in DR [110]. Administration of recombinant CTRP3 prevented BRB breakdown in STZ-induced diabetic models in vivo and preserved endothelial TJ integrity in HRECs under hyperglycemic stress by activating AMPK signaling, which enhanced the junctional expression of Cldn-5 and occludin, thereby reducing vascular leakage [111].

Persistent hyperglycemia also promotes the non-enzymatic glycation of proteins, lipids, and nucleic acids, leading to the formation of AGEs that alter the function of both the intracellular and transmembrane proteins [112]. Our laboratory observed an upregulation of Cldn-5 expression in AGE-treated HRECs, which paradoxically compromised barrier integrity by reducing TEER [21]. AGE inhibitors, such as pyridoxamine and aminoguanidine, have interestingly been shown to attenuate BRB dysfunction [113,114]; however, they failed to slow the progression of DR in a large randomized clinical trial involving 960 patients with type-1 DM [115].

#### 4.1.3. Inflammatory Cytokine–Mediated Cldn Dysregulation in BRB Breakdown

Inflammatory cytokines add a further layer of BRB compromise in DR. In diabetic settings, tumor necrosis factor-alpha (TNF-α) disrupts RPE barrier integrity by downregulating Cldn-19 and reducing TEER, thereby increasing paracellular permeability in polarized RPE monolayers [116]. Similarly, in HRECs, IL-6 trans-signaling drives endothelial permeability through STAT3 phosphorylation, nuclear factor kappa B (NF-κB) activation, and subsequent induction of inflammatory mediators. Selective inhibition of this pathway prevents TJ disassembly and BRB leakage [117]. Thus, cytokine-mediated BRB breakdown in DR targets both the oBRB (via Cldn-19 loss in RPE) and the iBRB (via IL-6–driven TJ disassembly without specific Cldn modulation).

#### 4.1.4. Hypoxia and VEGF Regulation of Cldns and Junctional Integrity

DR is often characterized by increased microvascular permeability and retinal neovascularization [118]. In the neural vascular system, including brain endothelial cells (bEND.3) and retinal microvessels in vivo, hypoxic exposure markedly reduced Cldn-5 protein levels, disrupted its junctional localization, decreased TEER values in vitro, and increased permeability to small molecular tracers in vivo (Figure 2), while Cldn-5 mRNA expression remained unchanged [119]. Mechanistically, hypoxia promotes caveolin-1-mediated endocytosis and autophagic degradation of Cldn-5, thereby reducing its availability at the junctional membrane [120]. Furthermore, hypoxia induces the expression of VEGF-A, a potent angiogenic factor that promotes vascular proliferation, permeability, and disruption of the iBRB in DR [121]. In support of this, prolonged exposure of iBRECs to VEGF-A_165_ increased Cldn-5 expression, peaking at day 6, although its membrane localization was markedly reduced compared with controls. By day 9, Cldn-5 levels normalized, yet barrier dysfunction persisted, suggesting that the impairment did not stem from altered protein abundance but from disrupted localization or assembly of Cldn-5 [122]. These findings indicate that sustained VEGF exposure induces barrier breakdown independent of continued VEGF signaling, driven instead by enduring structural alterations in endothelial junctions.

Advanced IRs exhibit chronic inflammation, pathological neovascularization, and profound iBRB disruption, which can ultimately progress to TRD in late stages [123]. In these settings, Cldn-5 disruption is a hallmark of increased vascular permeability and macular edema [95]. In the oxygen-induced retinopathy (OIR) model, which reproduces ischemic and hyperpermeability features of DR, both our laboratory and others have reported overexpression of Cldn-5 at both the mRNA and protein levels, along with upregulation of Cldn-2 [57,124]; this elevated expression did not correlate with improved barrier function as anticipated. Instead, Cldn-5 was aberrantly localized to cytosolic or non-junctional regions of the plasma membrane, suggesting that mislocalization, rather than depletion, underlies the observed increase in vascular permeability. In ROP, oxygen fluctuations during retinal vascular development disrupt Cldn expression, resulting in immature barrier formation and predisposing the retina to pathological angiogenesis and vascular leakage, constituting an early, developmentally sensitive pathogenic event. (Table 2).

#### 4.1.5. Extracellular Vesicle–Mediated Cldn Dysregulation in Barrier Dysfunction

Recent evidence identifies extracellular vesicles (EVs) as crucial mediators of intercellular communication and vascular dysfunction in DR [125]. These lipid bilayer-bound nanoparticles, including exosomes (30–150 nm), microvesicles (100–1000 nm), and apoptotic bodies (1000–5000 nm), are released by most cell types and transport proteins, lipids, and nucleic acids reflective of their cellular origin and state [126]. Vitreous samples from diabetic patients exhibit significantly elevated levels of Cldn-5-positive EVs compared with non-diabetic controls [127]. Exposing THP-1 macrophages to these vesicles induced the expression of pro-inflammatory cytokines such as TNF-α and IL-1β, suggesting that Cldn-5–containing EVs may contribute to retinal inflammation and barrier compromise (Figure 2). Proteomic profiling further revealed that these vesicles possess an endothelial-derived cargo consistent with ongoing vascular injury and blood–retinal barrier disruption [127]. Additionally, plasma-derived exosomes have been revealed to activate the classical complement pathway by binding C1q, inducing complement deposition, inflammation, and endothelial barrier breakdown in DR [128].

#### 4.1.6. oBRB Dysfunction and RPE Cldns

**Figure 3:**
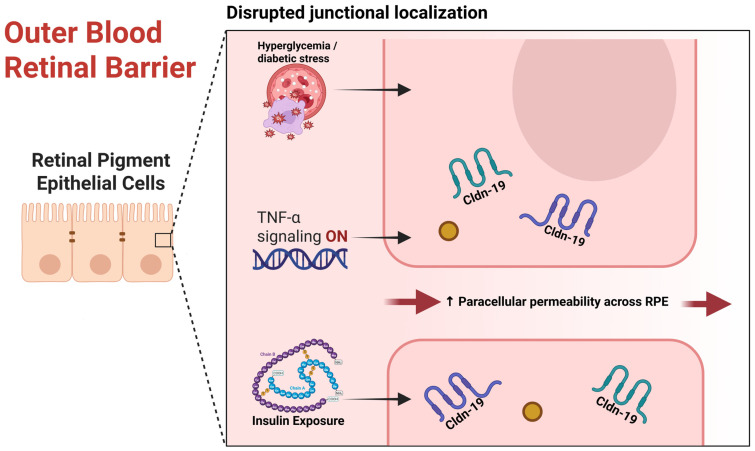
Mechanism of outer blood–retinal barrier breakdown in diabetic retinopathy. Created in BioRender. Salah, M. (2026) https://BioRender.com/33h7axi (accessed on 19 February 2026).

Evidence of oBRB disruption in ischemic retinopathies, particularly DR, is becoming increasingly recognized, and methods are being developed to identify and evaluate macromolecular leakage across the RPE in diabetic and ischemic animal models [129]. In diabetic settings, both in vivo and in cultured RPE cells, a study revealed that hyperglycemia and diabetic stress significantly plummeted Cldn-19 expression and disrupted its localization, resulting in elevated paracellular permeability. These findings implicate Cldn-19 dysfunction as a critical mechanism underlying oBRB compromise in DR [130]. In STZ-induced DR and OIR rodent models, significant macromolecule leakage through the oBRB was observed, with smaller molecules passing more readily than larger ones, making the number of severe oBRB leakage sites inversely correlated with macromolecule size. oBRB compromise was also accompanied by depletion of RPE-specific occludin, indicating disruption of TJ integrity [131]. Interestingly, in a transgenic zebrafish model expressing Cldn-19 tagged with enhanced green fluorescent protein in the RPE (RPE65: Cldn-19-eGFP), insulin-treated larvae exhibited RPE disruption, evident by junctional breakdown and mislocalization of Cldn-19, compromising the oBRB integrity. Similar results were observed in vitro using primary porcine RPE cells. Treatment with insulin reduced Cldn-19 expression and decreased the TEER, confirming loss of barrier function [132]. These findings reveal a novel mechanism by which insulin, independent of hyperglycemia, can trigger oBRB breakdown by impairing RPE-specific Cldn-19 (Figure 3). Additionally, proteomic analyses of ARPE-19 cells under HG revealed alterations in the resultant secreted proteins associated with cytoskeleton-linked adhesion/junction, including galectin-3-binding protein and moesin. These differentially secreted proteins were further validated in plasma from type-2 diabetic retinopathy patients in comparison to healthy donors, suggesting that RPE-altered secretome may contribute to oBRB compromise in DR [133]. Altogether, these findings underscore the need for deeper mechanistic studies, employing robust in vivo RPE-barrier models to assess how RPE breakdown contributes to the retinal vascular permeability in DR. (Table 2).

#### 4.1.7. Less-Studied Cldn Isoforms and Future Directions

Although multiple Cldns are expressed in the retinal vasculature, research on isoforms, such as Cldn-1, Cldn-3, Cldn-10, and Cldn-12 in DR, is limited, despite evidence that these proteins contribute to barrier selectivity and ion permeability in other tissues [4,134]. In DR, Cldn dysregulation is best viewed as an early and progressive pathogenic contributor rather than a sole downstream consequence. Hyperglycemia-induced metabolic stress, inflammation, and VEGF signaling directly disrupt Cldn expression and localization, leading to early BRB compromise that precedes overt microvascular pathology. To conclude, Cldn-5 remains the primary focus in DR due to its endothelial localization and essential role in maintaining iBRB integrity. Future investigations employing cell-specific and spatial approaches are needed to determine whether alterations in these lesser-studied Cldns also participate in BRB breakdown during DR progression.

### 4.2. Age-Related Macular Degeneration

Age-related macular degeneration (AMD) is a progressive degenerative disease of the central retina (macula) and is the leading cause of central irreversible vision impairment in older adults worldwide [135]. The complex pathophysiology of AMD is characterized by the accumulation of extracellular deposits known as drusen, accompanied by chronic inflammation and oxidative stress, which culminate in RPE dysfunction and photoreceptor degeneration [136]. Consistent with the contribution of oxidative stress and inflammation to RPE dysfunction in AMD, recent experimental studies have explored therapeutic strategies aimed at mitigating these pathogenic processes. For example, nanotherapeutic formulations combining resveratrol and metformin have been reported to enhance retinal penetration and exert prolonged protective effects in experimental models of macular degeneration, highlighting the translational potential of targeting upstream inflammatory and oxidative pathways in AMD [137].

AMD is clinically classified into two major forms: the non-neovascular (dry), characterized by geographic atrophy, and the neovascular (wet) type, marked by choroidal neovascularization (CNV). Both stages are fundamentally driven by the structural and functional failure of the tightly regulated ocular barriers [136]. Barrier breakdown in AMD contributes differentially to disease progression in each subtype: in dry AMD, impaired oBRB and RPE atrophy accelerate photoreceptor loss, whereas in wet AMD, disruption of TJ proteins facilitates choroidal endothelial invasion through Bruch’s membrane, promoting pathologic neovascularization and exudation [138].

RPE-barrier dysfunction is a key feature of AMD, particularly in advanced stages, where RPE cells undergo profound morphological and functional changes, including epithelial-to-mesenchymal transition (EMT) [139,140] EMT has been identified as a critical driver of blood–retinal barrier (BRB) breakdown in AMD and can be partially reversed by inhibition of TGF-β signaling [141]. In end-stage disease, extensive loss of RPE cells and photoreceptors culminates in geographic atrophy [142].

#### 4.2.1. Cldn Dysregulation and oBRB Failure

**Figure 4:**
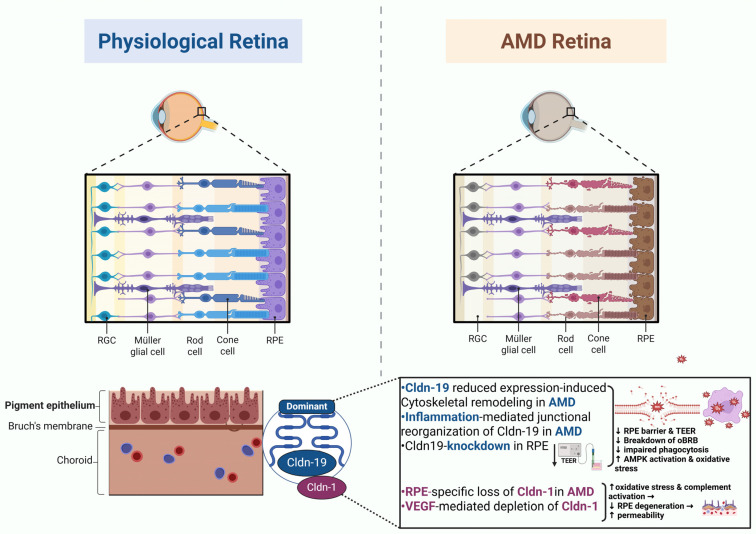
Pathogenesis of blood–retinal barrier breakdown in age-related macular degeneration. Created in BioRender. Salah, M. (2026) https://BioRender.com/y93psqp (accessed on 23 February 2026).

Although direct evidence linking specific Cldn isoforms to AMD pathogenesis remains limited, emerging studies in human RPE cells and animal models support a model in which relevant Cldn dysregulation contributes to oBRB failure in AMD. For instance, Cldn-19 mRNA levels are approximately 25-fold higher than Cldn-3 and several thousand-fold greater than other detectable Cldns in human fetal RPE [68]. Experimental silencing of Cldn-19 using small interfering RNA (siRNA) completely abolishes transepithelial resistance (TER) in cultured RPE cells, indicating a complete loss of barrier integrity [68]. Exposing Cldn-19-rich human RPE monolayers to pro-inflammatory cytokines (TNF-α, IL-1β, IFN-γ) markedly reduced the barrier resistance of the monolayer by over 80%, despite minimal or no change in Cldn-19 protein or mRNA levels. This finding suggests that cytokine-induced barrier dysfunction is driven by junctional reorganization and cytoskeletal remodeling rather than transcriptional downregulation of Cldn-19 [143]. Cldn-5 also plays a critical role in maintaining RPE integrity, as chronic suppression of Cldn-5 in mice fed a cholesterol-enriched diet led to pronounced RPE atrophy, while targeted knockdown of it in the macula of nonhuman primates induced localized RPE degeneration. These findings emphasize that proper Cldn-5 expression and intracellular trafficking are essential for oBRB stability and that disturbances in its regulation can precipitate RPE dysfunction and retinal pathology [86]. Cldn-1 deficiency in the RPE, however, leads to early and intermediate AMD-like changes in mice. RPE-specific loss of Cldn-1 has been reported to disrupt TJ integrity, markedly reduce barrier resistance, and result in barrier breakdown. This structural defect triggers oxidative stress, complement activation, macrophage infiltration, and mitochondrial dysfunction, culminating in RPE degeneration, basal deposit formation, and photoreceptor disorganization [67] (Figure 4).

#### 4.2.2. Cldn Dysregulation: Secondary Amplifiers of AMD

Overall, Cldn dysregulation in AMD is more likely to be a secondary pathogenic event that amplifies disease progression rather than a primary initiating factor. Oxidative stress, chronic inflammation, and iron dysregulation appear to precede and drive alterations in RPE composition, which in turn aggravate barrier breakdown, photoreceptor vulnerability, and choroidal dysfunction. However, whether early, subclinical Cldn alterations contribute to disease susceptibility remains an open question.

Collectively, Cldns and their junctional proteins reflect retinal barrier integrity in AMD. Measuring specific isoforms may help monitor iBRB or oBRB function and disease progression. Cldn-1, Cldn-19, and Cldn-5 show compartment-specific roles that link barrier dysfunction to AMD pathogenesis (Table 2).

### 4.3. Retinal Detachment and Traumas

Retinal detachment (RD) and ocular trauma represent acute, vision-threatening conditions that trigger profound structural and molecular disruptions of the BRB. Both pathologies involve physical separation of the neural retina from the underlying RPE [144,145], triggering a cascade of hypoxia, inflammation, and cellular stress responses that alter TJ integrity. Among TJ components, Cldn isoforms exhibit dynamic modulation during these events, leading to secondary barrier breakdown, edema, and photoreceptor degeneration [146].

#### 4.3.1. Classification of Retinal Detachment

RD is classified into three types: rhegmatogenous (RRD), caused by a mechanical retinal break; tractional (TRD), resulting from vitreoretinal scar tissue contraction; and exudative (ERD), characterized by subretinal fluid accumulation without a retinal tear, each featuring distinct mechanisms of BRB failure [147]. RRD is the most prevalent form and is associated with vascular leakage, which tends to improve after reattachment [148]. In chronic RRD, however, persistent hypoperfusion leads to RPE atrophy [149], while sustained BRB breakdown facilitates microglial activation and infiltration of peripheral immune cells into the retinal parenchyma, amplifying inflammation and tissue damage [150].

#### 4.3.2. TJ Disruption and Cldn Modulation in RD

Early studies using human and animal models demonstrated rapid disorganization of TJ proteins, including occludin, ZO-1, and Cldns, within hours of detachment [151,152,153]. Immunohistochemical analyses showed that Cldn-3 and Cldn-19, normally present at the apical TJ belt, become discontinuous or mislocalized to the cytoplasm after RD [154], which correlates with decreased TEER and increased paracellular leakage across RPE monolayers. These alterations underline the structural fragility of the oBRB following detachment.

Emerging evidence also implicates Cldn-17, an unexplored anion-selective TJ protein, as a critical regulatory factor in iBRB integrity. Cldn-17 deficiency in the retinas of OIR mice exacerbated vasoobliteration, neovascularization, and vascular leakage, positioning it as a potential upstream therapeutic target for IRs and TRD [72]. Similarly, Cldn-19 disruption activates cellular stress pathways: siRNA-mediated knockdown in human induced pluripotent stem cell (iPSC)-derived RPE cells can activate AMPK and trigger oxidative stress responses, such as elevated metallothionein expression and superoxide dismutase activity. This suggests the role of Cldn-19 as a structural sensor, initiating widespread stress signaling and potentially influencing RPE pathological processes such as migration and proliferation associated with proliferative vitreoretinopathy (PVR) following RRD [64] (Table 2).

Beyond structural alterations, transcriptomic profiling of retinal samples from patients with RRD revealed two interconnected molecular responses: a strong inflammatory and immune activation, characterized by upregulation of complement, microglial, leukocyte, and cytokine signaling genes, such as TNF and IL-6, and concurrent activation of photoreceptor death and stress pathways involving apoptosis, oxidative stress, and metabolic dysregulation. Pathway analysis indicated that early innate immune activation likely amplifies secondary photoreceptor degeneration [155].

In conclusion, RD-associated Cldn dysregulation represents an interesting molecular event that links BRB breakdown to inflammation and photoreceptor loss. Alterations or downregulation of Cldn-3, Cldn-5, Cldn-17, and Cldn-19 disrupt both the iBRB and the oBRB, expanding permeability and tissue injury. Future studies should delineate whether targeted modulation of specific Cldn isoforms can preserve junctional integrity and mitigate vision loss in RD and related retinal disorders.

**Table 2:** Summary of retinal disease states and associated Cldn alterations.

Disease State	Cldn Subtype	Alteration Type	Pathological Outcome/Effect	References
Diabetic Retinopathy	Cldn-5	Loss, mislocalization, or paradoxical upregulation.	Compromised endothelial integrity, increased vascular leakage, and BRB disruption.	[21,106,107,108]
	Cldn-19	Expression significantly plummeted and localization disrupted.	Breakdown of the outer BRB (oBRB) in diabetic settings.	[130]
	Cldn-19	Insulin-induced junctional breakdown and mislocalization (zebrafish RPE65)	TEER reduction, impaired RPE barrier integrity, and oBRB leakage	[132]
Age-related Macular Degeneration	Cldn-1	RPE-specific loss/ deficiency.	Disrupted TJ integrity, oxidative stress, complement activation, leading to RPE degeneration.	[67]
	Cldn-5	Chronic suppression/ knockdown.	Pronounced RPE atrophy and degeneration (oBRB instability).	[86]
	Cldn-19	Junctional reorganization/ cytoskeletal remodeling due to inflammatory cytokines.	Reduced RPE barrier resistance (a hallmark of AMD).	[138,140,143]
Retinal Detachment	Cldn-3, Cldn-19	Discontinuous or mislocalized to the cytoplasm after RD.	Decreased RPE monolayer resistance (TEER) and increased paracellular leakage (oBRB breakdown).	[154]
Ischemic Injury/ROP	Cldn-5	Overexpression but aberrant localization (cytosolic).	Increased vascular permeability and macular edema (iBRB failure).	[57,95,124]
	Cldn-17	Loss or deficiency.	Exacerbated vasoobliteration, neovascularization, and permeability.	[72]

### 4.4. Glaucoma and Optic Neuropathies

Glaucoma is characterized by progressive optic nerve head cupping and selective loss of retinal ganglion cells (RGCs) and their axons, ultimately resulting in irreversible visual field deficits [156,157]. Clinically, glaucoma is broadly classified into two major types: open-angle glaucoma (OAG) and angle-closure glaucoma (ACG). Epidemiological data indicate that OAG exhibits the highest prevalence in African populations, whereas ACG predominates in Asian populations. Global projections estimate that the number of individuals aged 40–80 years affected by glaucoma was approximately 64.3 million in 2013, rose to 76.0 million by 2020, and is expected to reach 111.8 million by 2040 [158]. Although elevated intraocular pressure (IOP) remains the primary risk factor for both pathologies, accumulating evidence implicates vascular dysregulation, inflammation, and oxidative stress in disease progression [159,160].

A critical yet often underrecognized element in this pathophysiology is the integrity of the BRB, which depends heavily on TJ proteins, including members of the Cldn family, to maintain ionic homeostasis and protect neural tissue from circulating toxins and immune factors.

#### 4.4.1. BRB Breakdown and Cldn-5 Dysregulation

Recent investigations utilizing the chronic ocular hypertension (COH) rat model to study how sustained IOP elevation affects the iBRB and contributes to RGC loss demonstrated that elevated IOP led to significant leakage of Evans blue dye into retinal tissue, indicating BRB breakdown. Biochemical analysis revealed marked downregulation and mislocalization of both Cldn-5 and occludin and was accompanied by increased retinal expression of VEGF inflammatory mediators (TNF-α, IL-1β) and by apoptotic loss of RGCs. Importantly, treatment with minocycline, an antioxidant and anti-inflammatory compound, restored Cldn-5 and occludin expression, reduced BRB leakage, and attenuated RGC apoptosis [161], suggesting a causal relationship between TJ disruption and neuronal degeneration.

Complementing these findings, a compelling preclinical study of a COH mouse model reported a downregulation of Cldn-5 expression in eyes with ocular hypertension (OHT) relative to controls, endothelial mitochondrial dysfunction, and subsequent leakage of the iBRB preceding RGC loss. Furthermore, AAV-mediated overexpression of Cldn-5 markedly reduced vascular leakage, preserved RGC density, and rescued visual behavior. These data further substantiate the concept that modulation of Cldn-dependent TJs in the cerebral-retinal microvascular endothelium is not only correlative but also causative in optic neuropathy pathogenesis [162]. Similar results were observed in ex vivo organotypic cultured mouse retinas exposed to elevated hydrostatic pressure, which confirmed that IOP-induced mechanical stress compromises TJ integrity at iBRB through downregulation and redistribution of Cldn-5 [163]. It thereby bridges the gap between biomechanical injury and molecular barrier dysfunction, suggesting that pressure-driven TJ alterations occur even in the absence of systemic inflammation.

#### 4.4.2. Cldns in Optic Nerve Vulnerability and Metabolic Stress

Disruption of Cldn-5 also exposes RGC axons to metabolic and immune stress. In a rat model, treatment with borneol, a monoterpenoid compound known to enhance BBB permeability, induced reversible downregulation and disorganization of Cldn-5 and occludin within optic nerve microvessels, leading to increased vascular permeability. These findings collectively suggest that Cldn-5 modulation critically determines optic nerve susceptibility to pressure- or drug-induced injury [164]. Beyond endothelial regulation, Cldn-11, the principal TJ component of oligodendrocyte myelin sheaths, plays a critical role in optic nerve physiology and glial barrier integrity. Mice lacking Cldn-11 exhibit disrupted axonal conduction and increased vulnerability to demyelinating injury [165]. Given that glaucomatous optic neuropathy involves progressive axonal degeneration and myelin remodeling, altered Cldn-11 expression may exacerbate axonal vulnerability within the optic nerve, though the mechanism remains to be fully elucidated.

#### 4.4.3. Cldns as Neuroprotective Targets

In summary, Cldn-11 emerges as a key regulator of vascular and glial barrier integrity in glaucomatous and ischemic optic neuropathies. Future studies should define diverse Cldn dynamics across disease stages and explore TJ-stabilizing therapies as neuroprotective interventions.

### 4.5. Retinitis Pigmentosa and Other Inherited Retinal Degenerations

Retinitis pigmentosa (RP) and other inherited retinal degenerations (IRDs) comprise a genetically diverse group of retinal disorders caused by mutations in over 80 genes affecting photoreceptor structure, function, or metabolism. These diseases are characterized by progressive degeneration of rod photoreceptors followed by secondary cone loss, leading to night blindness, limited visual fields, and eventual central vision impairment [166]. Although photoreceptor apoptosis stands as the principal pathogenic event, mounting evidence indicates that disruption of retinal barrier integrity contributes to disease progression and secondary neuroinflammation [167].

Histological and molecular studies in RP reveal early impairment of both the oBRB and iBRB [168]. For instance, the RPE, forming the oBRB, is an active participant in intraocular inflammation. Investigations revealed that human RPE cells robustly respond to inflammatory cytokine stimulation, particularly interleukin-1β (IL-1β) and TNF-α by upregulating IL-6 mRNA expression and secretion in a time- and dose-dependent manner [169]. Therefore, sustained inflammatory stimulation and RPE degeneration subsequently disrupt the Cldn-rich TJ complex [154], facilitating paracellular leakage and immune infiltration.

In rd10 mice, a model of autosomal recessive RP (Pde6b mutation), progressive photoreceptor loss was accompanied by discontinuous and patchy localization of Cldn-5 staining, reduced protein expression, and vascular leakage at the iBRB, coinciding with microglial activation and Müller glial proliferation. Furthermore, vascular remodeling and both iBRB and oBRB breakdown become evident by postnatal day P35–P40 in this model, when rods are largely lost, but reductions in retinal perfusion occur earlier, suggesting that vascular dysfunction precedes overt degeneration [170]. Comparable hypoperfusion is observed in RP patients [171,172], and systemic hyperoxia mitigates photoreceptor loss in both animal models [173,174] and patients with RP [175], indicating that insufficient blood flow and Cldn-5–linked barrier impairment actively contribute to photoreceptor decline.

Collectively, these data position Cldn-5 and Cldn-19 as pivotal molecular determinants of retinal barrier stability in IRDs. In RP, inflammatory cytokines, hypoxia, and metabolic stress converge to destabilize the RPE and vascular TJs, amplifying microglial activation and photoreceptor death. However, restoration of TJ homeostasis preserves BRB integrity and delays neurodegeneration. These findings underscore the therapeutic potential of targeting Cldn signaling to mitigate retinal barrier failure and secondary neuronal loss in hereditary retinopathies.

## 5. Regulation of Cldn Function in the Retina

The organization of TJ complexes in the retina hinges on the finely tuned expression, trafficking, or assembly of the Cldn family members, predominantly Cldn-5 in vascular endothelium and Cldn-19 along with Cldn-3 and Cldn-1 in the RPE. These proteins undergo both transcriptional regulation and post-translational modifications (PTMs) that determine their abundance, localization, and barrier function. Disruption of these regulatory mechanisms contributes to barrier leakage in ischemic and inflammatory retinal diseases (Table 3).

### 5.1. WNT/β-Catenin Signaling and Norrin

The WNT/beta-catenin pathway plays a fundamental role in maintaining blood-tissue barrier integrity. Norrin, a secreted ligand essential for retinal vascular development, can activate the WNT/*β*-catenin signaling pathway by binding to Frizzled-4 (FZD_4_) and low-density lipoprotein-related receptors (LRP5/6), forming a complex that has been shown to be of major importance for both BBB and BRB [85]. In fact, absence of norrin/FZD_4_ signaling severely impairs retinal capillary growth [85]. While LRP5 knockout *(Lrp5−/−)* mice exhibit persistent hyaloid vasculature and regressed retinal capillaries [176], FZD_4_ deletion during adulthood reduced the expression of Cldn-5, leading to BRB breakdown, whereas blocking its function via anti-FZD_4_ monoclonal antibody similarly induced a BRB dysfunction [177].

### 5.2. Non-Coding RNAs and Epigenetic Regulation

Beyond classic transcription factors, non-coding RNAs (ncRNAs), including microRNAs (miRNAs), long non-coding RNAs (lncRNAs), and circular RNAs (circRNAs), serve as vital elements of epigenetic regulation that control gene expression and TJ function [178]. Under both HG conditions in vitro and in diabetic mice, miR-200 and miR-466 expression was significantly upregulated in endothelial cells, which correlated with a reduction in Cldn-5 translation without altering mRNA abundance, resulting in loss of Cldn-5 protein level, disassembling TJ, increasing paracellular permeability, and reducing TEER, a mechanism relevant to DR [179]. Although these findings were derived from vascular and dermal endothelial models rather than retinal tissue, the mechanism bears direct relevance to the molecular pathology of DR, where Cldn-5 dysregulation similarly contributes to BRB breakdown. Further investigation is warranted to determine whether comparable miRNA-mediated modulation of Cldn-5 occurs in retinal endothelium under hyperglycemic stress.

### 5.3. Post-Translational Modifications of Cldns

PTMs represent critical regulatory checkpoints that dictate the acute stability of the BRB structure in response to external signals, such as inflammation or growth factors [180]. Aberrant phosphorylation is one of the distinct mechanisms of barrier failure in DR. Normally, PKCζ facilitates TJ formation and assembly [181]; however, in type 2 diabetic models, it undergoes hyperactivation and phosphorylation, causing mislocalization of key scaffold proteins, particularly occludin, disrupting the TJ architecture, and causing increased leakage of plasma proteins into the retina [182,183]. A significant nuance observed in experimental models of DR and OIR is the association between the iBRB and PTMs, rather than a quantifiable loss of core TJs, such as Cldns or occludin, as evident by the upregulation of both Cldn-2 and Cldn-5 in OIR [57,124], confirming that the acute phase of iBRB failure is driven by hyperactive kinase signaling acting on associated proteins, rendering them structurally incompetent despite their normal presence at the cell surface.

Inflammatory cytokines further exacerbate this dysfunction. Transforming growth factor-β1 (TGF-β1), the multifunctional cytokine involved in immune modulation, inflammation, and fibrosis [184], provides another direct link between disease pathology and Cldns PTM. TGF-β1 has been reported to increase paracellular permeability in retinal endothelial cells by promoting the tyrosine phosphorylation of Cldn-5 and VE-cadherin [185]. Tyrosine phosphorylation is often a signal for protein endocytosis and degradation, meaning this PTM directly results in the functional downregulation and loss of junctional integrity. Likewise, Rho kinase (ROCK1), a major downstream effector of the RhoA cytoskeletal remodeling pathway, has been shown to directly phosphorylate Cldn-5 and occludin in brain endothelial cells [186]. Pharmacological inhibition of ROCK1 effectively counteracted VEGF-induced inflammatory signaling, restored Cldn-5 expression and membrane localization, and enhanced its transcription in both Kimba diabetic mice and HRECs [187].

### 5.4. Cldn Isoform-Specific Roles

In summary, while multiple Cldn isoforms contribute to retinal barrier function, Cldn-5 dominates mechanistic and translational interest given that it is the principal endothelial Cldn at the iBRB, highly responsive to vascular stressors such as hyperglycemia, VEGF, and inflammation, and directly regulated by major signaling cascades. Other Cldns, such as Cldn-19 and Cldn-3, play specialized epithelial roles in the RPE. Future research exploring their signaling, post-translational control, and compensatory dynamics will be essential to broaden therapeutic strategies beyond the Cldn-5-centric paradigm currently dominating retinal barrier biology (Table 3).

## 6. Cldns as Therapeutic Targets in the Retina

Given that the expression and junctional incorporation of the Cldn family determine both iBRB and oBRB function, Cldns are deemed to be promising therapeutic targets. Emerging intervention strategies can be broadly classified into three complementary domains: (1) pharmacological stabilization or restoration of Cldn function to reinforce barrier tightness; (2) gene-based augmentation of Cldn expression through viral or CRISPR-mediated delivery; and (3) the development of advanced delivery systems capable of efficiently reaching the retinal endothelium or RPE.

### 6.1. Pharmacological Modulation of Cldn-5

Therapeutic modulation of Cldn-5 expression has been employed to transiently and selectively open the iBRB, thereby enhancing retinal drug delivery, a major obstacle due to the iBRB’s restricted permeability to macromolecules and hydrophilic agents. siRNA-mediated knockdown of Cldn-5 in retinal capillary endothelium induces a reversible, size-selective increase in barrier permeability, permitting controlled passage of impermeable molecules [100,188]. Beyond transient barrier opening, pharmacologic strategies aimed at stabilizing Cldn-5 expression and localization have shown promise in preserving BRB integrity. In particular, Norrin effectively reverses VEGF-induced hyperpermeability in both in vitro and in vivo diabetic models [189] through activation of the FZD4 receptor and its downstream effector Disheveled-1 (DVL-1), which directly interacts with and stabilizes Cldn-5 at endothelial junctions [190]. Basigin, another regulator of TJ complexes, mediates VEGF- and TNF-α–induced mislocalization of Cldn-5 in diabetic retinas; conversely, siRNA silencing of basigin restores proper Cldn-5 junctional localization and barrier integrity both in vitro and in vivo [191,192]. Similarly, Fingolimod (FTY720), an immunomodulator acting on sphingosine-1-phosphate receptors (S1PRs), preserved the expression of Cldn-5, ZO-1, and occludin in STZ-induced diabetic rats, thereby protecting against diabetes-associated retinal barrier disruption [103]. Furthermore, triciribine, a small molecule with anti-angiogenic properties, ameliorated the pathological effects of Cldn-5 overexpression in the OIR model, restoring normal vascular architecture and barrier selectivity [22]. Dexamethasone further reverses the inflammatory insults by inhibiting TNF-α–induced NF-κB activation in high-glucose and inflammatory retinal models, thereby preserving Cldn5 expression and BRB function [183].

### 6.2. Modulation of RPE Claudins: Cldn-3 and Cldn-19

Modulation of both Cldn-3 and Cldn-19, although still emerging, has shown promise in reducing paracellular permeability and partially restoring native RPE characteristics, thereby reinforcing the oBRB. For instance, overexpressing both Cldns in ARPE-19 cells, a cell line that expresses all TJ proteins except for the Cldn family, increased TEER, reduced leakage of small ions and polyethylene glycol tracers, and reestablished the epithelial phenotype that is typically lost with continuous passaging. The presence of these Cldns has also influenced cellular proliferation and migration and activated downstream signaling pathways such as Akt, shifting the cells toward a more native RPE phenotype [193]. A follow-up study further confirmed that even when Cldn-3 and Cldn-19 are overexpressed in ARPE-19, they can still regulate RPE-specific gene expression without fully integrating into the apical junctional complex. Cldn-19, in particular, modulated steady-state levels of ADAM9 and tyrosinase and promoted a more mature RPE-like phenotype [193], suggesting that Cldn-19 is implicated in signaling and gene regulatory roles more than just a structural barrier protein. To date, research on Cldn-19 remains largely mechanistic and confined to RPE cell lines or iPSC-derived cells, with no reported therapeutic studies in animal models, including safety, delivery, timing, or long-term efficacy.

The success of voretigene neparvovec (AAV2-hRPE65v2) in restoring visual function in patients with RPE65-mediated retinal dystrophy highlights the clinical feasibility of subretinal AAV-based gene therapy, paving the way for similar strategies aimed at modulating Cldn expression (e.g., Cldn-5 or Cldn-19) to restore barrier integrity in IRs [194].

### 6.3. Gene-Based Therapeutic Strategies

Recent advances in CRISPR/Cas9 gene editing have revolutionized ocular therapeutics, demonstrating precise and robust gene modulation within the retinal tissues. Vectors such as AAV2 and AAV8, already validated for subretinal delivery, could be engineered for endothelial- or RPE-specific editing of Cldn genes to restore barrier function. Moreover, CRISPR activation (CRISPRa) or CRISPR interference (CRISPRi) systems could precisely upregulate or suppress Cldn expression without inducing double-strand breaks, offering a safer approach for fine-tuning paracellular permeability in diseases characterized by barrier dysfunction [195].

### 6.4. Delivery Challenges and Emerging Solutions

Despite these advances, retinal drug and gene delivery continue to face multifactorial hurdles. The iBRB/oBRB themselves impede systemic access to therapeutics. While intravitreal and subretinal routes bypass such barriers, they entail procedural risks and often require repeated administration for small molecules or AAV. Emerging delivery technologies offer opportunities: lipid nanoparticles (LNPs) and other nanocarriers enable nucleic acid delivery (siRNA, mRNA) to retinal cells; EV-based or targeted peptide carriers may achieve cell-specific uptake; and biodegradable sustained-release implants can reduce dosing frequency. Each approach must balance transduction efficiency, immunogenicity (AAV, CRISPR), payload stability, and local toxicity [196,197,198,199]. While these strategies have not yet been applied to directly modulate Cldns in the retinal vasculature, their successful use in retinal gene delivery and endothelial gene regulation highlights their potential as future therapeutic modalities, suggesting that effective restoration of barrier function in retinal disease will likely require combined strategies: acute pharmacologic stabilization (steroids/anti-VEGF or pathway modulators) to limit edema and inflammation, followed by longer-term genetic or epigenetic approaches to correct Cldn expression/trafficking where appropriate. Prioritizing preclinical studies that link Cldn restoration to meaningful preservation of retinal function will be essential before clinical translation.

## 7. Conclusions and Future Perspectives

Cldns are central to retinal barrier integrity. In multiple retinal pathologies, altered Cldn expression or mislocalization, especially Cldn5 in the iBRB and Cldn19 in the RPE, is a consistent feature of vascular leakage and neurodegeneration. Cldns function not only as structural components but also as sensors of metabolic, oxidative, and mechanical stress. Through these roles, they shape the balance between barrier stability and breakdown. This understanding highlights new therapeutic opportunities in which selective modulation of specific Cldn isoforms could restore controlled permeability while preserving normal exchange.

Several gaps remain. The contributions of less-studied Cldns, such as Cldn-2, Cldn-10, and Cldn-17, in regulating vascular permeability are still unclear, as are their interactions with Cldn5 or Cldn19. The post-translational sites that govern Cldn turnover during disease also need to be defined. How iBRB and oBRB Cldns coordinate their responses to systemic stressors, including diabetes and aging, remains an open question. There is also interest in whether retina-targeted CRISPRa or AAV tools can achieve durable, isoform-specific expression without vascular effects in other tissues, and whether soluble or vesicle-associated Cldn fragments in ocular fluids could serve as non-invasive measures of BRB health.

Integrating multi-omics data with advanced imaging may yield early biomarkers of Cldn dysfunction and allow detection of barrier failure before irreversible vision loss. As the field moves forward, Cldns are likely to be viewed not only as structural gatekeepers but also as actionable molecular targets for restoring vascular and epithelial integrity in retinal disease.

## Figures and Tables

**Table 1:** Cldn subtypes expressed in different retinal layers/cell types.

Retinal Layer/Cell Type	Cldn Subtype(s) Expressed	Role/Function	References
oBRB RPE Cells	Cldn-1, Cldn-3	Contribute to the structural integrity of the oBRB and regulate RPE gene and protein expression independently of barrier function.	[65,66,67]
	Cldn-10	Expressed at low levels and may play supportive or region-specific roles.	[66]
	Cldn-19	Principal structural and functional Cldn of the oBRB and integral to RPE barrier robustness and phagocytic capacity.	[45,64,68]
iBRB Endothelial Cells	Cldn-1	An essential component of functional tight junctions in retinal endothelial cells.	[69]
	Cldn-5	Predominant structural component and molecular gatekeeper of the iBRB, essential for selective permeability.	[4,52,70,71]
	Cldn-17	Emerging role as a critical regulatory factor in iBRB integrity (anion-selective TJ protein).	[72]
Müller Glia	Cldn-1, -3, -19	Generally absent mammalian retina, though reported in teleost fish endfeet.	[73,74]

**Table 3:** Mechanisms of Cldn regulation under pathologic conditions.

Pathological Condition/ Stress Factor	Cldn Target	Regulatory Mechanism/ Pathway Involved	Outcome on Barrier Function	References
Diabetic Stress/Hyperglycemia	Cldn-5	AMPK signaling activation (by CTRP3) and AGEs effects.	AMPK activation, increased expression, and reduced leakage. High glucose and AGEs upregulate expression but compromise barrier integrity by mis-localization.	[21,111,112]
Ischemia/ Hypoxia	Cldn-5	Caveolin-1 (Cav-1)-mediated endocytosis and autophagic degradation.	Reduced Cldn-5 protein, loss of junctional localization, and increased permeability (iBRB failure).	[119,120]
Angiogenesis/Sustained VEGF	Cldn-1	Prolonged VEGF exposure.	Markedly depleted from the plasma membrane, strongly correlating with increased permeability.	[69]
	Cldn-5	Sustained VEGF exposure (via mechanisms independent of continued VEGF signaling).	Increased Cldn-5 expression but marked reduction in membrane localization (disrupted assembly), leading to persistent barrier dysfunction.	[122]
Inflammation/cytokines	Cldn-19	TNF-α, IL-1β, and IFN-γ acting through junctional and cytoskeletal reorganization.	Significantly reduced RPE barrier resistance.	[143]
	TJ proteins (in general)	IL-6 trans-signaling drives permeability through STAT3 phosphorylation and NF-κB activation.	TJ disassembly and BRB leakage.	[155,170]
Cellular Stress (Pathologic)	Cldn-1, Occludin	Endoplasmic Reticulum (ER) stress (in RPE cells).	Enhanced mRNA and protein expression, epithelial barrier resistance (compensatory tightening).	[88]
	Cldn-5	Endoplasmic Reticulum (ER) stress (in endothelial cells).	Downregulated expression and vascular impairment (iBRB failure).	[87]
	Cldn-19	Knockdown/Disruption.	Triggers the activation of AMPK and oxidative stress responses.	[64]

## Data Availability

No new data were created or analyzed in this study.

## Abbreviations

The following abbreviations are used in this manuscript:

Abbreviation	Definition
AAV	Adeno-associated virus
ACG	Angle-closure glaucoma
AGE	Advanced glycation end-product
AMD	Age-related macular degeneration
AMPK	AMP-activated protein kinase
BBB	Blood–brain barrier
bEND.3	Brain endothelial cell line clone 3
BRB	Blood–retinal barrier
BREC	Bovine retinal endothelial cell
Cldn	Claudin
CNV	Choroidal neovascularization
COH	Chronic ocular hypertension
CTRP3	C1q/tumor necrosis factor-related protein 3
DVL-1	Disheveled-1
DR	Diabetic retinopathy
EC	Endothelial cell
ECL1/2	Extracellular loop 1/2
ER	Endoplasmic reticulum
ERD	Exudative retinal detachment
EV	Extracellular vesicle
FHHNC	Familial hypomagnesemia with hypercalciuria and nephrocalcinosis
FZD4	Frizzled-4 receptor
H&E	Hematoxylin and eosin
HG	Hyperglycemia/High glucose
HREC	Human retinal endothelial cell
ICAM-1	Intercellular adhesion molecule-1
IFN-γ	Interferon-gamma
IL	Interleukin
ILM	Inner limiting membrane
INL	Inner nuclear layer
IOP	Intraocular pressure
IR	Ischemic retinopathy
IRD	Inherited retinal degeneration
iBRB	Inner blood–retinal barrier
iBREC	Immortalized bovine retinal endothelial cell
JAM	Junctional adhesion molecule
LNP	Lipid nanoparticle
LPS	Lipopolysaccharide
LRP5/6	Low-density lipoprotein receptor-related protein 5/6
MAPK	Mitogen-activated protein kinase
miRNA	MicroRNA
NF-κB	Nuclear factor kappa-light-chain-enhancer of activated B cells
NO	Nitric oxide
NPDR	Non-proliferative diabetic retinopathy
NVU	Neurovascular unit
OAG	Open-angle glaucoma
OIR	Oxygen-induced retinopathy
OLM	Outer limiting membrane
ONL	Outer nuclear layer
oBRB	Outer blood–retinal barrier
PDR	Proliferative diabetic retinopathy
PDZ	Postsynaptic density protein domain
PKC	Protein kinase C
POS	Photoreceptor outer segment
PTM	Post-translational modification
PVR	Proliferative vitreoretinopathy
RD	Retinal detachment
RGC	Retinal ganglion cell
RhoA	Ras homolog family member A
RNA-seq	RNA sequencing
ROCK	Rho-associated kinase
ROP	Retinopathy of prematurity
RP	Retinitis pigmentosa
RPE	Retinal pigment epithelium
RT-qPCR	Reverse transcription quantitative polymerase chain reaction
S1P	Sphingosine-1-phosphate
S1PR	Sphingosine-1-phosphate receptor
siRNA	Small interfering RNA
SQSTM1 (p62)	Sequestosome 1
STZ	Streptozotocin
TEER	Trans-epithelial/-endothelial electrical resistance
TGF-β	Transforming growth factor-beta
TJ	Tight junction
TNF-α	Tumor necrosis factor-alpha
TRD	Tractional retinal detachment
VE-cadherin	Vascular endothelial cadherin
VEGF	Vascular endothelial growth factor
Wnt	Wingless-related integration site
W/D	Wet-to-dry ratio
ZO-1/ZO-2/ZO-3	Zonula occludens-1/-2/-3
